# The effects of multiwalled carbon nanotubes and *Bacillus subtilis* treatments on the salt tolerance of maize seedlings

**DOI:** 10.3389/fpls.2022.1093529

**Published:** 2022-12-09

**Authors:** Ying Luo, Wenzhi Zeng, Guoqing Lei, Yaling Hou, Chang Ao, Haorui Chen, Thomas Gaiser, Amit Kumar Srivastava

**Affiliations:** ^1^ State Key Laboratory of Water Resources and Hydropower Engineering Science, Wuhan University, Wuhan, China; ^2^ State Key Laboratory of Simulation and Regulation of Water Cycle in River Basin, China Institute of Water Resources and Hydropower Research, Beijing, China; ^3^ Crop Science Group, Institute of Crop Science and Resource Conservation (INRES), University of Bonn, Bonn, Germany

**Keywords:** antioxidant enzyme, ionic balance, maize growth, osmolytes, salt stress

## Abstract

Nanomaterials, including multiwalled carbon nanotubes (MWCNTs), have been recently applied in agriculture to improve stress resistance, leading to contradictory findings for antioxidant responses and mineral nutrient uptake. A pot experiment involving maize in low-salinity sandy loam soils was conducted with the application of different concentrations (0, 20, 50 mg/L) of MWCNTs and the growth-promoting rhizobacterium *Bacillus subtilis* (*B. subtilis*). The dose-dependent effects of MWCNTs were confirmed: 20 mg/L MWCNTs significantly promoted the accumulation of osmolytes in maize, particularly K^+^ in the leaves and roots, increased the leaf indoleacetic acid content, decreased the leaf abscisic acid content; but the above-mentioned promoting effects decreased significantly in 50 mg/L MWCNTs-treated plants. We observed a synergistic effect of the combined application of MWCNTs and *B. subtilis* on plant salt tolerance. The increased lipid peroxidation and antioxidant-like proline, peroxidase (POD), and catalase (CAT) activities suggested that MWCNTs induced oxidative stress in maize growing in low-salinity soils. *B. subtilis* reduced the oxidative stress caused by MWCNTs, as indicated by a lower content of malondialdehyde (MDA). The MWCNTs significantly increased the leaf Na^+^ content and leaf Na^+^/K^+^ ratio; however, when applied in combination with *B. subtilis*, the leaf Na^+^/K^+^ ratio decreased sharply to 69% and 44%, respectively, compared to those of the control (CK) group, the contents of which were partially regulated by abscisic acid and nitrate, according to the results of the structural equation model (SEM). Overall, the increased osmolytes and well-regulated Na^+^/K^+^ balance and transport in plants after the combined application of MWCNTs and *B. subtilis* reveal great potential for their use in combating abiotic stress.

## 1 Introduction

Thirty-three percent of irrigated farmland and twenty percent of arable land in the world have been affected by salinization (Mustafa et al., 2019). Soil salinization adversely affects plant growth and has become one of the major limiting factors for crop productivity worldwide (Sarma et al., 2012). Several methods have been used to ameliorate soil salinity and to improve the salt stress tolerance of various plant species, such as irrigation and soil leaching, chemical amelioration, and the use of genetically modified plants or salt-resistant varieties (Chu et al., 2016; Hafeez et al., 2021). However, the high cost, technical requirements, consumption of resources, labor intensiveness, and potential environmental hazards have relatively constrained their application.

Nanoparticles (NPs) are particles of matter that exist at the nanoscale level, ranging from 1 to 100 nm in size (Shweta et al., 2017). Multiwalled carbon nanotubes (MWCNTs) are carbon-based NPs that are relatively environmentally safer than metal NPs. The adsorption and penetration capabilities of MWCNTs make them unique carriers for fertilizers and agrochemicals (Chhipa and Joshi, 2016). The hypertonic and high-sodium environmental conditions observed under salt stress usually prevent plants from easily taking up water and nutrients. Studies have shown that MWCNTs have the potential to improve nutrient use efficiency and alleviate macro- and micronutrient deficiencies in plants (Shang et al., 2019; Hu et al., 2021). MWCNTs interact with nutrients through hydrogen bonding, surface tension, viscous forces, or transient dipole interactions in the soil (Tiwari et al., 2013; Monreal et al., 2015; Shang et al., 2019). Moreover, MWCNTs can act as nutrient microcarriers that enter plants through pores within the cell wall and membrane or ion channels or *via* endocytosis to promote water and nutrient absorption (Mastronardi et al., 2015; Monreal et al., 2015). The physicochemical state and morphology of plants also change in the microenvironment where MWCNTs and plants coexist. Seddighinia et al. (2019) stated that MWCNTs increase the uptake of water and nutrients because of changes in the vascular system structure in plant stems and roots through the promotion of the elongation of shoots and roots. According to recent reports, MWCNTs affect the activities of key enzymes involved in regulating nitrogen and carbon metabolism, resulting in the accumulation of plant carbohydrates (sugars and starch, soluble proteins, and nitrogen) (Hu et al., 2021). In addition, MWCNTs may act as plant growth-promoting compounds to increase plant biomass under salt stress (Vithanage et al., 2017). Zhao et al. (2019) also reported that MWCNTs enhance nitrate reductase-dependent nitric oxide biosynthesis, maintain the ionic and oxidative balance, and promote plant growth under salt stress.

As a tool to guarantee food security under both climate change and various ecological constraints that is cost-effective and environmentally friendly, MWCNTs, which can be used in small amounts and thus are efficient, may be used as convenient tools to increase crop yields and ensure the sustainability of land production (Shang et al., 2019). However, several reports have been released describing the concentration-dependent effects of MWCNTs on plant growth. Generally, optimal concentrations of MWCNTs promote growth, but excessive concentrations may exert negative or even toxic effects. For example, Mondal et al. (2011) reported that a low concentration of MWCNTs (23 mg/L) effectively improved the germination rate of mustard seeds, promoted root and shoot elongation, accelerated the water absorption of seeds, and improved the stability of the cell membrane, while a higher concentration (46 mg/L) inhibited seed water absorption, germination, and embryo development. Similarly, adverse effects of high concentrations of MWCNTs (100 mg/L) on the agronomic traits, photosynthesis, and antioxidant enzyme activities of basil under salt stress have also been reported, which was proposed to result from oxidative stress caused by the aggregation of MWCNTs in the plants (Gohari et al., 2020). However, Lin and Xing (2007) found no biological toxicity of high-dose treatments (up to 2000 mg/L MWCNTs) in various crop species. Similar to other NPs, the size, shape, concentration, and surface functionalization of MWCNTs play crucial roles in their application (Hatami et al., 2017). Some effective new strategies must be developed to avoid the possible adverse effects of NPs. Most notably, some attempts to promote plant growth through microbial-nanomaterial composites have been reported in recent years. The development of nanocomposites containing active *Bacillus thuringiensis* has further improved the efficacy and shelf life of pesticides and reduced the required dose (Koul, 2019). Furthermore, Ahmed et al. (2021) used *Azotobacter salinestris ASM* strains that were resistant to metal NPs to circumvent the toxic effect of NPs through extracellular polymer-mediated chelation and biomass adsorption, reducing the absorption of metal NPs by roots and increasing the seed germination rate, photosynthetic activity, biomass, and yield.

*Bacillus subtilis* (*B. subtilis*), a widely used environmentally friendly plant growth-promoting rhizobacterium (PGPR) that tolerates high-temperature, high-pH, and hyperosmotic conditions (Ashwini and Srividya, 2014), is widely present in nature and synthesizes bioactive substances, such as antimicrobial and insecticidal components, siderophores and chelators (Saikia et al., 2018), and exopolysaccharides (Upadhyay et al., 2011). The potential application of *B. subtilis* for salt resistance and plant growth promotion has also been documented (Sarma et al., 2012). *B. subtilis* can increase nutrient availability and the uptake of plant macronutrients such as potassium, nitrogen, and phosphorus (Ramesh et al., 2014) through nitrogen fixation and phosphate or potassium solubilization (Li et al., 2021). By secreting phytohormones such as indoleacetic acid (IAA), abscisic acid (ABA) and gibberellins (GAs) (Idris et al., 2004) and by increasing osmolyte contents and antioxidant enzyme activities, *B. subtilis* has been shown to significantly improve plant stress resistance.

The combined application of *B. subtilis* and MWCNTs has recently been targeted for pollutant removal (Hartono et al., 2018), but the potential of this approach for plant growth promotion and stress protection has not been investigated. As mentioned previously, MWCNTs have been reported to enhance nutrient or ion absorption by plants, but contrasting results and opinions have been reported concerning the antioxidant response and accumulation of important ions, namely, Na^+^ and K^+^, in plants under salt stress (Martinez-Ballesta et al., 2016; Zhao et al., 2019). Focusing on agronomic traits, osmolytes, lipid peroxidation and antioxidant activities, and nutrient and ion absorption of plants, we applied *B. subtilis* and different concentrations of MWCNTs separately or in combination to maize plants under salt stress to explore the potential independent and combined effects of these treatments on the salt tolerance of maize.

## 2 Results

### 2.1 Agronomic traits

*B. subtilis*, MWCNTs, and their combination increased the maize dry/fresh weight and plant height to different degrees. As shown in **Table 1**, compared with that of the control (CK) group, the dry weight of the M20 group (addition of 20 mg/L MWCNTs) increased by approximately 20.0%, with no significant changes in fresh weight or plant height; the dry/fresh weights or plant height of the M50 group (addition of 50 mg/L MWCNTs) didn’t change significantly compared with those in the CK group. The maize in the BS (*B. subtilis*) and BM20 (addition of 20 mg/L MWCNTs with *B. subtilis*) treatment groups presented similar agronomic traits, including plant height and dry/fresh weight, and the last two parameters increased by approximately 30% and 15%, respectively. The plants in the BM50 group (addition of 50 mg/L MWCNTs with *B. subtilis*) presented the largest dry/fresh weight and were the tallest. Compared with those in the CK group, the dry/fresh weights and plant height of the BM50 group increased significantly by approximately 26.5%, 32.5%, and 8.9%, respectively.

**Table 1:** Effects of *B. subtilis* and MWCNTs on maize agronomic traits, osmolyte contents, and endogenous hormone contents under salt stress.

Treatment	Dry weight (g/plant)	Fresh weight (g/plant)	Plant height (cm)	Nitrate-nitrogen (μg/g FW)	Soluble sugars (mg/g FW)	IAA (μg/g FW)	ABA (μg/g FW)
CK	0.40±0.07c	2.87±0.08d	39.96±1.10b	58.42±1.07c	30.59±0.2a	3.93±0.08bc	16.71±0.23a
M20	0.48±0.02ab	2.97±0.15cd	40.97±1.01ab	67.22±0.85b	28.19±0.36b	4.21±0.04a	13.71±0.23d
M50	0.45±0.02bc	3.20±0.07bcd	39.79±1.05b	59.98±0.94c	28.81±0.34b	3.83±0.06c	13.48±0.31d
BS	0.52±0.03a	3.27±0.03bc	40.98±1.00ab	72.32±0.98a	31.23±0.23a	4.04±0.06b	14.82±0.21c
BM20	0.51±0.03ab	3.37±0.07ab	41.88±0.98ab	69.97±0.78a	30.24±0.44a	3.61±0.06d	14.40±0.24c
BM50	0.53±0.03a	3.63±0.19a	43.52±1.00a	70.38±1.04a	28.90±0.47b	4.07±0.05ab	15.83±0.14b
*B. subtilis*	20.90***	20.95***	5.11*	134.91***	6.62*	3.66	4.21*
MWCNTs	1.41	5.15*	0.76	8.16**	20.32***	0.84	28.34***
*B. subtilis* × MWCNTs	1.78	0.02	1.22	18.03***	4.11*	30.80***	42.72***

The dry/fresh weight data are presented as the means of three replicates. The plant height data are presented as the means of 20 replicates ± SE and were compared using Duncan’s multiple range tests. The nitrate-nitrogen, soluble sugar, IAA and ABA contents are presented as the means of 7 replicates ± SE and were compared using Duncan’s multiple range tests. Within each column, the values with the same lowercase letter are not significantly different. Significant levels of the two-way analysis of variance are denoted *P < 0.05, **P < 0.01, ***P < 0.001. The treatments were as follows: CK, no addition of MWCNTs or *B. subtilis*; M20, addition of 20 mg/L MWCNTs without *B. subtilis*; M50, addition of 50 mg/L MWCNTs without *B. subtilis*; BS, addition of *B. subtilis* without MWCNTs; BM20, addition of 20 mg/L MWCNTs with *B. subtilis*; and BM50, addition of 50 mg/L MWCNTs with *B. subtilis*.

### 2.2 Osmolytes

Compared with that in the CK group, the nitrate-nitrogen contents of the plants from the M20, BS, BM20, and BM50 treatment groups increased significantly (*P* < 0.05) (by approximately 15.1%, 23.8%, 19.8%, and 20.5%, respectively), but not the M50 treatment group. As shown in **Table 1**, the interaction between *B. subtilis* and MWCNTs exerted a significant effect on the nitrate-nitrogen content (*P* < 0.001). The soluble sugar contents in the plants receiving the M20, M50, and BM50 treatments were significantly lower (by approximately 10%) than those in the CK group, and no significant differences were observed between the BS, BM20, and CK treatments (**Table 1**, P < 0.05). After the combined application of MWCNTs with *B. subtilis*, the content of soluble sugars increased compared with that in plants treated with MWCNTs alone; for example, the soluble sugar content in the plants from the BM20 group was significantly greater than that in plants from the M20 group (**Table 1**, P < 0.05). According to the two-way analysis of variance, the interaction between *B. subtilis* and MWCNTs exerted a significant effect on the contents of proline (**Supplementary Table 1**, P < 0.001) and soluble sugars (**Table 1**, P < 0.05).

### 2.3 Endogenous hormones

**Table 1** shows the plant ABA and IAA contents following the different treatments. Compared with that in the CK group, the ABA content in the leaves of the plants receiving each treatment decreased significantly (*P* < 0.05), but the range of decrease was different. The ABA contents decreased by 18.0% and 19.3% in the M20 and M50 groups, respectively, when MWCNTs were applied alone, but they increased to some extent in the BS, BM20, and BM50 groups.

The IAA content in the leaves of the plants from the M20 group was significantly higher than that in the CK group, but the opposite result was observed for the BM20 group (**Table 1**, *P* < 0.05). No significant differences in IAA contents were observed between the other treatment groups and the CK group (**Table 1**, *P* < 0.05). As shown in **Table 1**, both ABA and IAA contents were significantly affected by the interaction of *B. subtilis* and MWCNTs (*P* < 0.001).

### 2.4 Lipid peroxidation and antioxidant activities

**Figure 1:**
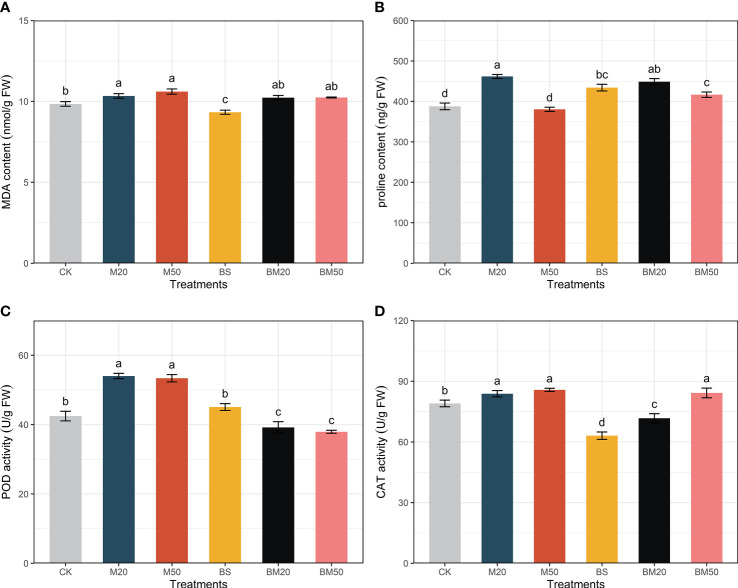
Effects of different treatments on lipid peroxidation and antioxidant activities. **(A)** MDA contents, **(B)** proline contents, **(C)** POD activity, and **(D)** CAT activity. The MDA and proline contents and POD and CAT activity data are presented as the means of five replicates ± SE and were compared using Duncan’s multiple range tests. Within each figure, the values with the same lowercase letter are not significantly different. The treatments were as follows: CK, no addition of MWCNTs or *B. subtilis*; M20, addition of 20 mg/L MWCNTs without *B. subtilis*; M50, addition of 50 mg/L MWCNTs without *B. subtilis*; BS, addition of *B. subtilis* without MWCNTs; BM20, addition of 20 mg/L MWCNTs with *B. subtilis*; and BM50, addition of 50 mg/L MWCNTs with *B. subtilis*.

MWCNTs increased the MDA content in maize plants, while *B. subtilis* inoculation reduced it (**Figure 1A**, *P* < 0.05). Coapplication of MWCNTs and *B. subtilis* reduced the MDA content compared with treatment with MWCNTs alone, although the difference was not significant (**Figure 1A**, *P* < 0.05).

The proline contents in the plants from the M20, BS, BM20, and BM50 groups were significantly higher than those in the CK group (**Figure 1B**, *P* < 0.05). The proline content in maize did not change significantly after the application of 50 mg/L MWCNTs alone compared with the CK group but increased significantly after MWCNTs were applied in combination with *B. subtilis* (**Figure 1B**, *P* < 0.05).

The M20 and M50 treatments increased CAT and POD activities in maize plants (**Figures 1C, D**, *P* < 0.05). The BS treatment did not significantly change POD activity but significantly reduced CAT activity (**Figures 1C, D**, *P* < 0.05). The BM20 and BM50 treatments both significantly decreased the POD activity in maize leaves (**Figure 1C**, *P* < 0.05). The BM20 treatment significantly decreased CAT activity, but the BM50 treatment significantly increased CAT activity in maize leaves (**Figure 1D**, *P* < 0.05).

### 2.5 Na^+^ and K^+^ absorption and distribution

**Figure 2:**
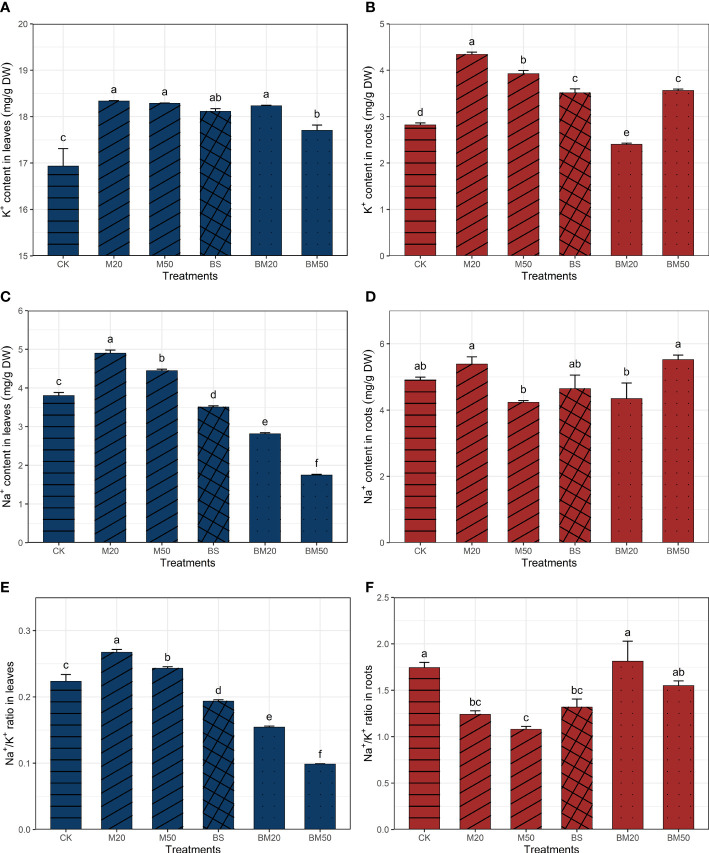
Effects of different treatments on the Na^+^ and K^+^ contents and the Na^+^/K^+^ ratio. **(A)** Leaf K^+^ contents, **(B)** root K^+^ contents, **(C)** leaf Na^+^ contents, **(D)** root Na^+^ contents, **(E)** leaf Na^+^/K^+^ ratio, **(F)** and root Na^+^/K^+^ ratio. The Na^+^ and K^+^ contents and the Na^+^/K^+^ ratio data are presented as the means of three replicates ± SE and were compared using Duncan’s multiple range tests. Within each figure, the values with the same lowercase letter are not significantly different. The treatments were as follows: CK, no addition of MWCNTs or *B. subtilis*; M20, addition of 20 mg/L MWCNTs without *B. subtilis*; M50, addition of 50 mg/L MWCNTs without *B. subtilis*; BS, addition of *B. subtilis* without MWCNTs; BM20, addition of 20 mg/L MWCNTs with *B. subtilis*; and BM50, addition of 50 mg/L MWCNTs with *B. subtilis*.

The contents of Na^+^ and K^+^ in the leaves of each treatment group are shown in **Figure 2**. The K^+^ contents in the leaves of plants in the M20 and M50 groups were significantly higher than those in the CK group, and the content of Na^+^ and the Na^+^/K^+^ ratio were also significantly higher (**Figures 2A, C, E**, *P* < 0.05). The K^+^ contents in the leaves of plants in the BS, BM20, and BM50 groups were significantly higher than those in the CK group; however, the Na^+^ contents were significantly lower, resulting in significantly lower Na^+^/K^+^ ratios (**Figures 2A, C, E**, *P* < 0.05). In particular, the Na^+^/K^+^ ratio decreased significantly in the BM20 and BM50 groups (by approximately 31.3% and 56.0%, respectively) (**Figure 2E**, *P* < 0.05).

Compared with those in the CK group, the root K^+^ contents in the plants from the M20, M50, BS, and BM50 treatment groups increased by approximately 54.0%, 39.2%, 24.5%, and 24.3%, respectively, but decreased in the BM20 treatment group. Significant differences in root Na^+^ contents were not observed between the plants in the CK and in each of the other treatment groups, but the root Na^+^ contents in the plants from the M20 and BM50 treatment groups were slightly higher than those in the CK group. The root Na^+^/K^+^ ratio of the plants in the M20, M50, and BS treatment groups was significantly lower than that in the CK treatment group, but no significant differences were observed between the BM20, BM50, and CK treatment groups (**Figure 2B, D, F**, *P* < 0.05).

### 2.6 Principal component analysis

**Figure 3:**
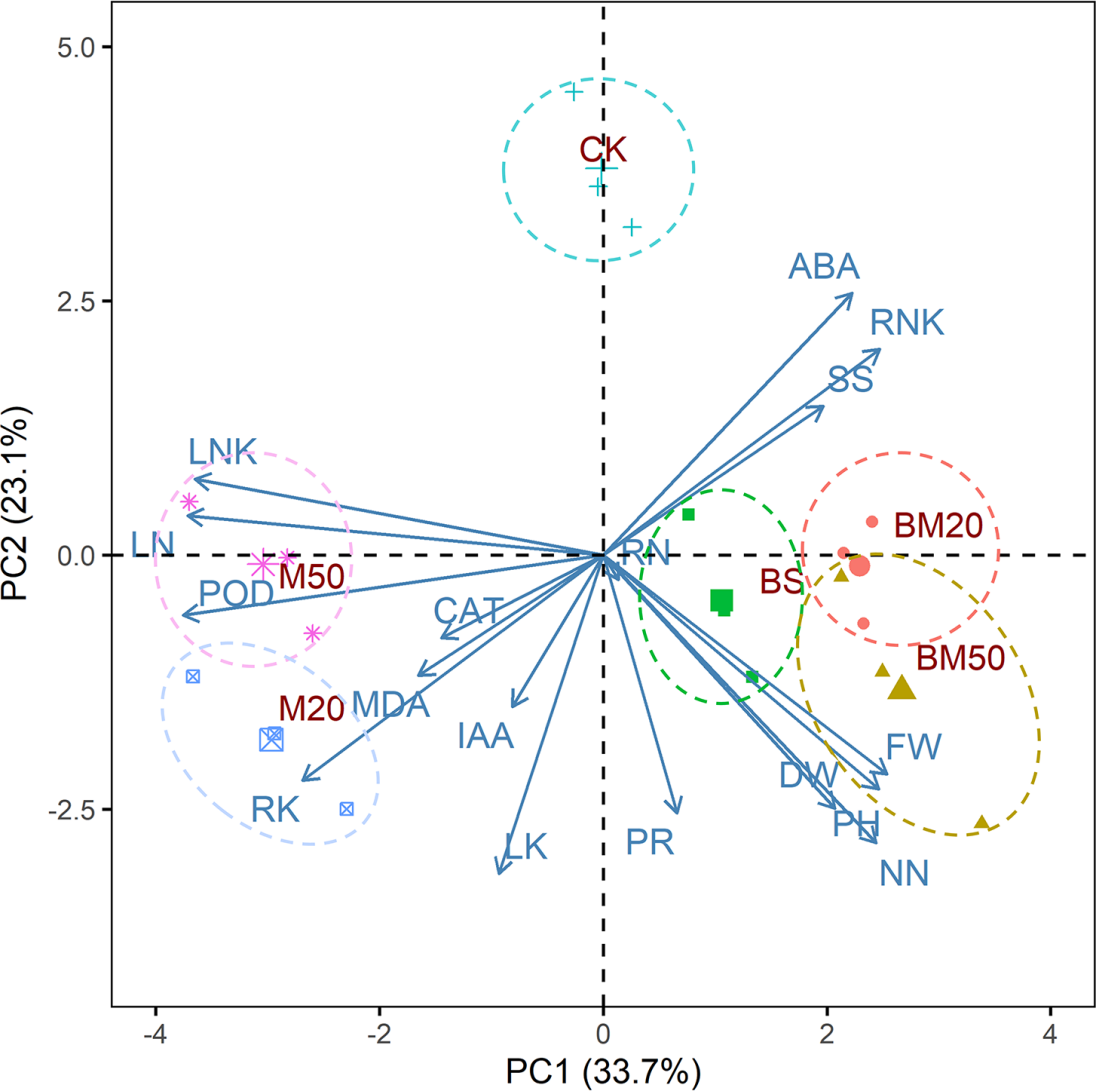
Results of the PCA of select parameters of plants receiving different amendment treatments. DW, FW, PH, NN, SS, ABA, RNK, PR, IAA, RN, POD, LN, LNK, LK, RK, MDA, and CAT represent dry weight, fresh weight, plant height, nitrate-nitrogen content, soluble sugar content, ABA content, root Na^+^/K^+^ ratio, proline content, IAA content, root Na^+^ content, POD activity, leaf Na^+^ content, leaf Na^+^/K^+^ ratio, leaf K^+^ content, root K^+^ content, and MDA content, respectively. The treatments were as follows: CK, no addition of MWCNTs or *B. subtilis*; M20, addition of 20 mg/L MWCNTs without *B. subtilis*; M50, addition of 50 mg/L MWCNTs without *B. subtilis*; BS, addition of *B. subtilis* without MWCNTs; BM20, addition of 20 mg/L MWCNTs with *B. subtilis*; and BM50, addition of 50 mg/L MWCNTs with *B. subtilis*.

As shown in **Figure 3**, the loadings of leaf Na^+^ content, POD content, leaf Na^+^/K^+^ ratio, root K^+^ content, nitrate-nitrogen content and agronomic traits for the first principal component were the largest. In addition to POD content and agronomic traits, they are all indicators that characterize the content and proportion of important inorganic ions in plants. The first principal component mainly characterized the information related to ionic balance. Leaf K^+^, nitrate-nitrogen, ABA content, root K^+^ content, proline content and agronomic traits constituted the largest loadings of the second principal component. In addition to ABA content and agronomic traits, they are all indicators that characterize the content of Inorganic and organic osmolyte in plants. The second principal component mainly represented information related to osmolyte. With respect to the first principal component, BM50 had the highest score, and M50 and M20 had the lowest scores. The BM50 treatment tended to reduce the leaf Na^+^/K^+^ ratio and enhance plant growth, whereas the M50 and M50 treatments exerted the opposite effects. Regarding the second principal component, compared with the CK treatment, the M50, M20, BS, BM20, and BM50 treatments tended to increase the osmolyte contents.

### 2.7 Correlation analysis and structural equation model analysis

**Figure 4:**
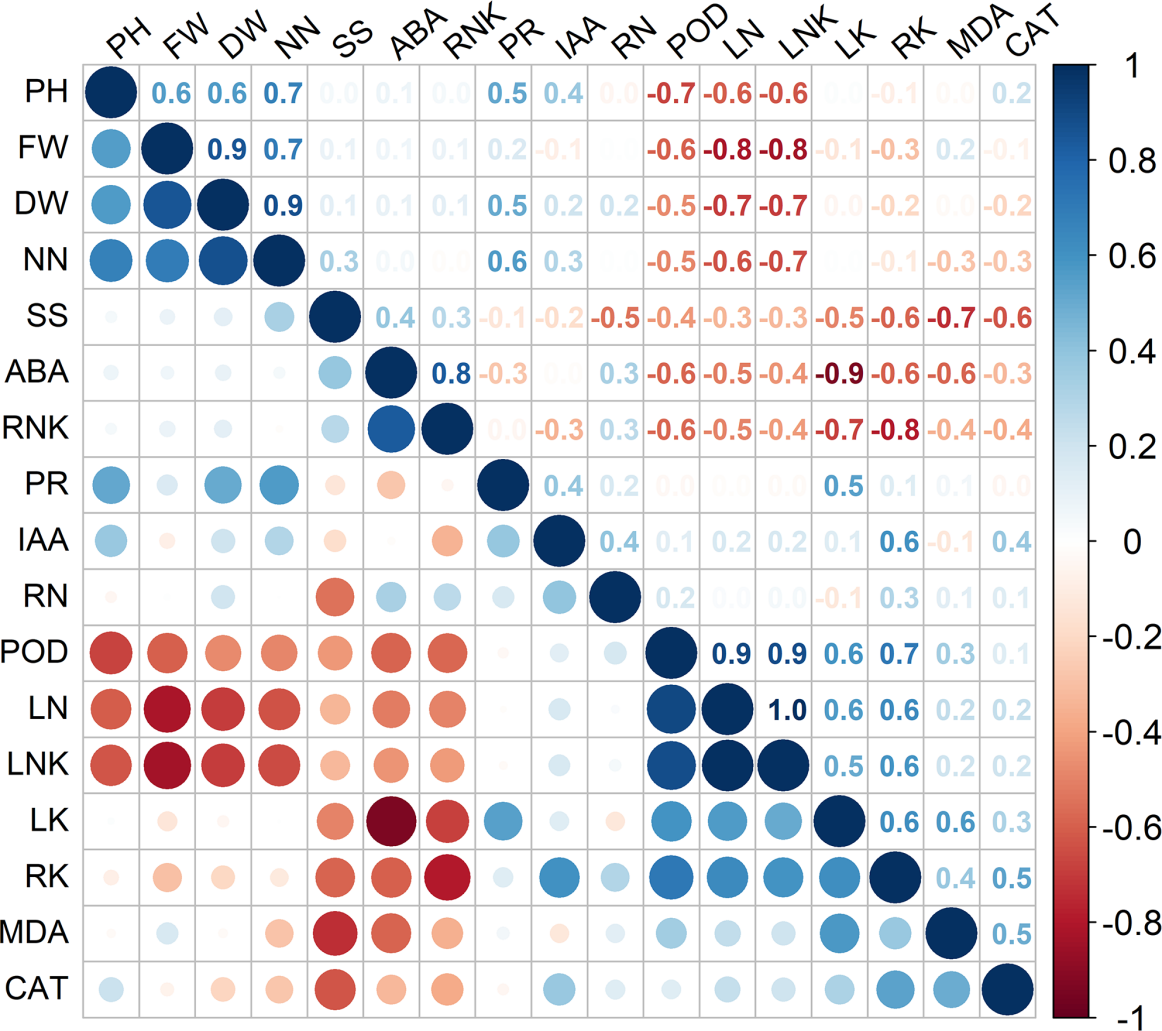
Results of Spearman’s correlation analysis of select parameters of plants receiving different treatments. DW, FW, PH, NN, SS, ABA, RNK, PR, IAA, RN, POD, LN, LNK, LK, RK, MDA, and CAT represent dry weight, fresh weight, plant height, nitrate-nitrogen content, soluble sugar content, ABA content, root Na^+^/K^+^ ratio, proline content, IAA content, root Na^+^ content, POD activity, leaf Na^+^ content, leaf Na^+^/K^+^ ratio, leaf K^+^ content, root K^+^ content, and MDA content, respectively.

As shown in **Figure 4**, significant positive correlations were observed among plant height, fresh weight, and dry weight (*P* < 0.05). The leaf Na^+^/K^+^ ratio was significantly negatively correlated with various agronomic traits, such as plant height and fresh/dry weight (**Figure 4**, *P* < 0.05). The nitrate-nitrogen content was not only significantly positively correlated with plant height and dry/fresh weight but also significantly negatively correlated with leaf Na^+^ content and the leaf Na^+^/K^+^ ratio (**Figure 4**, *P* < 0.05). Although the ABA content was significantly negatively correlated with the leaf K^+^ content, Na^+^ content, leaf Na^+^/K^+^ ratio, root K^+^ content, MDA content, and POD activity, it was significantly positively correlated with the root Na^+^/K^+^ ratio (**Figure 4**, *P* < 0.05). The proline content was significantly positively correlated with the nitrate-nitrogen content and K^+^ content, each of which is an important osmolyte (**Figure 4**, *P* < 0.05). POD activity was significantly positively correlated with the leaf K^+^ content, Na^+^ content, leaf Na^+^/K^+^ ratio, and root K^+^ content, but it was significantly negatively correlated with nitrate-nitrogen content, plant height and fresh/dry weight (**Figure 4**, *P* < 0.05).

**Figure 5:**
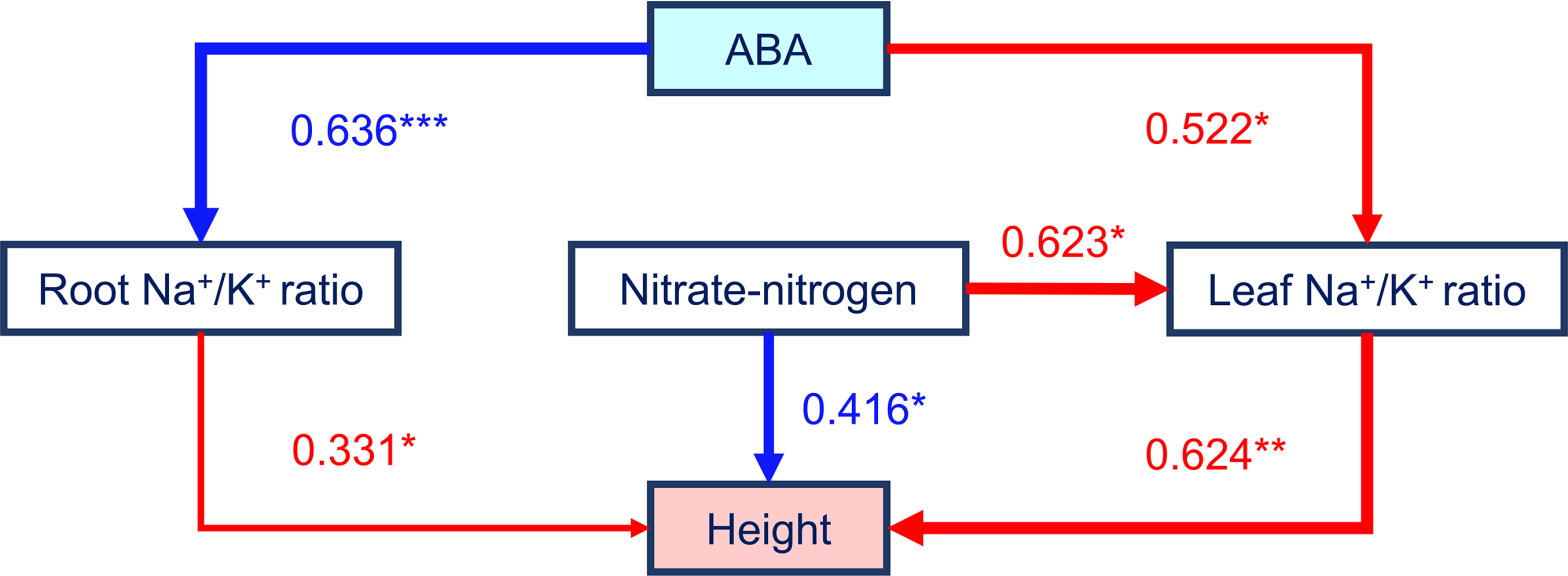
Effects of four variables on the height of maize plants growing in low-salinity soil, based on the SEM. The relationships among the plant height, leaf Na^+^/K^+^ ratio, root Na^+^/K^+^ ratio, nitrate-nitrogen content, and ABA content were explained by a SEM. The path coefficients are presented above the arrows. A thicker line indicates a stronger effect (* = p < 0.05, ** = p < 0.01, and *** = p < 0.001).

Three plant agronomic traits constituted large loadings of both the first and the second principal component according to the results of the PCA (**Figure 3**), and they showed significant correlations with many other parameters. Considering the results of our correlation analysis and PCA, plant height was selected as a proxy for maize growth and salt tolerance and as the dependent variable, and an SEM was constructed to analyze the mediating effects of the leaf Na^+^/K^+^ ratio, nitrate-nitrogen content, root Na^+^/K^+^ ratio, and ABA content on plant height. Our hypothesized SEM fit the data well (χ^2^ = 0.972, df = 3, χ^2^/df = 0.324, *P* = 0.808; comparative fit index (CFI) = 1.000; root mean square error of approximation (RMSEA) = 0.000). As shown in **Figure 5** and in **Table 2**, regarding the total standardized effect, the nitrate-nitrogen content was the strongest predictor of maize plant height under salt stress, with a total effect of 0.805, followed by the leaf Na^+^/K^+^ ratio (λ = -0.624) and root Na^+^/K^+^ ratio (λ = -0.331). The leaf Na^+^/K^+^ ratio had the greatest direct effect on plant height (λ = -0.624). The nitrate-nitrogen content exerted a direct positive effect on plant height and a direct negative effect on the leaf Na^+^/K^+^ ratio; thus, the nitrate-nitrogen content had the greatest total effect on plant height. On the one hand, ABA content exerted a direct negative effect on the leaf Na^+^/K^+^ ratio, which promoted plant growth; on the other hand, ABA content had a direct positive effect on the root Na^+^/K^+^ ratio, which inhibited plant growth. The total standardized effect suggests that ABA content has an overall positive effect on maize plant height, although the effect is rather small. Overall, the SEM showed that the nitrate-nitrogen content and the leaf Na^+^/K^+^ ratio were the most important drivers of maize plant height.

**Table 2:** The contribution of each variable to plant height based on the direct, indirect, and total effects according to the result of the SEM.

Parameters	Direct path	Indirect path	Total effect
leaf Na^+^/K^+^ ratio	-0.624	–	-0.624
nitrate-nitrogen	0.416	0.389	0.805
root Na^+^/K^+^ ratio	-0.331	–	-0.331
ABA	–	0.115	0.115

## 3 Discussion

### 3.1 MWCNTs and *Bacillus subtilis* improved maize osmoadaptation by increasing osmolyte levels

Salinity is one of the most important environmental constraints limiting land productivity. In the short term, salt stress first leads to osmotic stress, which reduces the moisture content; in the long term, salt stress causes a cytoplasmic nutrient imbalance in plants, an accumulation of sodium ions, and ionic stress, which subsequently result in yellowing and necrosis (Acosta-Motos et al., 2017). Plants have a variety of traits to combat salts in the soil solution. The most essential trait is osmotic adjustment; all cells must accumulate sufficient amounts of osmolytes to counterbalance the extra osmotic pressure in the soil solution and maintain turgor (Munns and Gilliham, 2015). Soluble sugars are important compatible osmolytes. The content of soluble sugars in maize leaves was slightly reduced in response to the application of MWCNTs (**Table 1**, *P* < 0.05). According to SAINAO et al. (2020), the soluble sugar content of rice roots exposed to 1000 mg/L MWCNTs (whose outer diameter was less than 30 nm) decreased significantly, which may reflect an oxidative response caused by the infiltration and blockage of MWCNTs in plant roots. Jiang et al. (2012) suggested that under the stimulation of the highest concentration of silver NPs, plant photosynthesis was inhibited, which led to a reduction in carbohydrate levels. *B. subtilis* has been reported to promote the accumulation of osmolytes in plants (Saleem et al., 2021), increase the activity of antioxidant enzymes (Medeiros and Bettiol, 2021), and reduce reactive oxygen species (ROS) accumulation in plants under salt stress. The combined use of *B. subtilis* and MWCNTs results in greater soluble sugar accumulation than the use of MWCNTs alone, suggesting the potential of *B. subtilis* to provide oxidative protection while stimulating the production of organic osmolytes.

Potassium and nitrate are important inorganic osmolytes that help maintain intracellular osmolarity, thereby enabling control of cell turgor pressure that is critical for cell expansion, stomatal movement, and pollen tube growth (Kroeger et al., 2011; Saito and Uozumi, 2019). In this experiment, the nitrate-nitrogen content in the plants in the M20, BS, BM20, and BM50 groups increased significantly (**Table 1**, *P* < 0.05). Moreover, *B. subtilis* exerted a very significant effect on the nitrate-nitrogen content in maize (**Table 1**, *P* < 0.001). It has been reported that *B. subtilis* could reduce the loss of soil nitrate-nitrogen (Sun et al., 2020; Yang et al., 2021), accelerate nitrate-nitrogen absorption, transport, and transformation (Nakano and Zuber, 1998), and improve its use rate (Rong-fa et al., 2019). Several studies have also shown that carbon NPs adsorb nitrogen in ammonia, release hydrogen ions, and facilitate the uptake of water and nutrients by plants (Wu et al., 2010; Vithanage et al., 2017). In the present study, two-way ANOVA revealed significant effects of MWCNTs on both root and leaf K^+^ contents (**Supplementary Table 1**). The potential of MWCNTs to promote water and ion uptake by plants has received increasing attention. MWCNTs have also been shown to promote K^+^ uptake by plants under saline stress and normal conditions (Martinez-Ballesta et al., 2016; Zhao et al., 2019; Joshi et al., 2020). Chen et al. (2020) performed a rectification of an ion current (RIC) assay, and found that carbon nanomaterials preferentially transport K^+^ to a greater extent than other cations in plant cells. The increase in nitrate accumulation in maize by *B. subtilis* and the promotion of K^+^ uptake in maize by MWCNTs are important mechanisms to improve the salt tolerance of maize, consistent with the SEM results (**Figure 5**).

Compared with that in M50, the greater accumulation of organic and inorganic osmolytes in M20 partially explains the increase in dry weight and plant height, and treatment with *B. subtilis* alone and in combination with MWCNTs increased the K^+^/nitrate uptake and proline accumulation, which helped maintain cell turgor pressure.

### 3.2 MWCNTs and *Bacillus subtilis* affected maize lipid peroxidation and antioxidant activities

According to **Figure 1A**, MWCNT-treated maize exhibited an increase in lipid peroxidation under saline stress, but *B. subtilis* released oxidative stress in maize plants, as indicated by the lower MDA content. Hong et al. (2022) observed increased MDA contents in MWCNT-treated cabbage, and Ghanbari et al. (2017) confirmed the dose-dependent toxicity of MWCNTs due to the increase in mitochondrial ROS formation. Inoculation with *B. subtilis* under saline stress conditions reduced plant MDA levels (Hidri et al., 2019).

Proline, a nonenzymatic antioxidant and one of the most high-affinity compatible osmolytes, is involved in quenching ROS and protecting membrane and protein structures (Anwar Hossain et al., 2014). In this study, 20 mg/L MWCNTs significantly increased the proline content in maize leaves, but the proline content decreased significantly to the CK level after treatment with an increased concentration of MWCNTs (**Figure 1B**, *P* < 0.05). Karami and Sepehri (2018) also observed a similar concentrate-dependent phenomenon in barley under salt stress. According to Rahmani et al. (2020), high concentrations of MWCNTs (up to 1000 mg/L) still promote proline synthesis in *Salvia miltiorrhiza* leaves under normal conditions. Different plant species and growth environments led to different results. *B. subtilis* significantly increased the proline content in maize, which was also significantly higher than that in the CK group after the bacteria were applied together with 50 mg/L MWCNTs (**Figure 1B**, *P* < 0.05).

The increased nonenzymatic and enzymatic antioxidants, and the increased MDA contents in our study suggested oxidative stress in maize caused by higher concentrations of MWCNTs under salt stress, which was alleviated by the application of *B. subtilis*, as indicated by the lower MDA contents. Hatami et al. (2017); Karami and Sepehri (2018), and Rahmani et al. (2020)also observed oxidative stress induced by MWCNTs in plants.

### 3.3 MWCNTs and *Bacillus subtilis* regulated maize ion balance

Our SEM showed that the contents of Na^+^, K^+^, nitrate, and ABA exerted significant direct or indirect effects on the height of maize plants under salt stress (**Figure 5**). Na^+^ toxicity is considered one of the main factors leading to cell death under salt stress, and K^+^ is an important ion to maintain electrolyte and osmotic balance; thus, plant cells must maintain a lower Na^+^/K^+^ ratio to resist salt stress (Kader et al., 2006). Leaves are the main sites of photosynthesis and other metabolic activities and are the main organs that experience the effects of Na^+^ toxicity (Zhang et al., 2018). The plants in the BS, BM20, and BM50 groups all showed significant decreases in the leaf Na^+^/K^+^ ratio (**Table 2**, *P* < 0.05). Significant changes in leaf Na^+^ and K^+^ contents were also observed by Saleem et al. (2021) and other researchers in their related studies on the increase plant salt tolerance induced by *B. subtilis*. In the M20 and M50 groups, both the leaf Na^+^ and K^+^ contents increased significantly. Martinez-Ballesta et al. (2016) suggested that more effective water uptake caused increased Na^+^ and K^+^ contents in MWCNT-treated plants. The root K^+^ content in the M20 and M50 groups significantly increased, but the root Na^+^ content did not change significantly. Some contradictory results were reported in previous studies. Zhao et al. (2019) observed a significant increase in the K^+^ content and a significant decrease in the Na^+^ content in the roots of 20 mg/L MWCNT-treated rape plants. However, Martinez-Ballesta et al. (2016) observed no significant change in the K^+^ content and a significant increase in the Na^+^ content in the roots of 10 mg/L MWCNT-treated broccoli plants. These differences in results are possibly due to differences in plant type and the concentration of applied MWCNTs.

According to the SEM, nitrate exerted a direct negative effect on the leaf Na^+^/K^+^ ratio. The application of a certain dose of nitrate promoted root Na^+^ efflux (Dai et al., 2015) and reduced Na^+^ accumulation in plants under salt stress (Rao and Sharma, 1995; Jaenicke et al., 1996). Nitrate, a substrate, participates in the process of nitric oxide-induced plant tolerance; as a result, this phenomenon reduces the absorption of Na^+^ by plants under salt stress and reduces the Na^+^/K^+^ ratio (Zhao et al., 2019). The uptake of soil K^+^ and nitrate by most plants is positively correlated and mutually promoted (Coskun et al., 2017). Some nitrate transporters expressed in plants, such as NRT1.1 (Fang et al., 2020) and NRT1.5 (Wang et al., 2004), are also involved in K^+^ transport.

The plant endogenous hormone ABA is an important signaling compound in the plant salt stress response. During the early stages of stress responses, ABA promotes plant survival by regulating the osmotic balance and ion absorption and closing stomata to reduce water loss, but the prolonged overaccumulation of ABA in the leaves of plants under salt stress inhibits plant growth (He and Cramer, 1996; Chen et al., 2001; Zhang et al., 2021). In the present study, the ABA content in the plant leaves decreased after the application of MWCNTs, consistent with the findings from a study by Zhang et al. (2017a). Low ABA levels are often maintained in the leaves of *B. subtilis*-treated plants (Barnawal et al., 2017; Woo et al., 2020; Akhtyamova et al., 2021). In our study, a significant negative correlation was observed between the ABA content and leaf/root K^+^ content (**Figure 4**, *P* < 0.001), and PCA revealed a close relationship between the K^+^ content and ABA content (**Figure 3**, *P* < 0.001). The change in the ABA content also exerted a significant effect on the leaf and root Na^+^/K^+^ ratios according to the SEM, suggesting that ABA is involved in the process of Na^+^ and K^+^ absorption, transport, and recycling. High-affinity potassium transporter (*HKT*) genes, whose expression is regulated by NaCl and ABA, are known to be involved in Na^+^ or K^+^ transport in higher plants (Shkolnik-Inbar et al., 2013; Zhang et al., 2019). **B. subtilis* GB03* emits a volatile signal that induces salt tolerance, and this signal induces increases the expression of the *HKT1* gene in *Arabidopsis* leaves and promotes Na^+^ transport from the shoots to the roots (Zhang et al., 2008).According to the results of our study, ABA might be a candidate for that signal. Among all the treatments, the BM50 treatment resulted in the maximum plant height and dry/fresh weight of maize, which is likely related to the decrease in the leaf Na^+^/K^+^ ratio in response to the increase in ABA and nitrate contents.

## 4 Materials and methods

### 4.1 Materials

**B. subtilis* AB 90008* was purchased from the China Center for Type Culture Collection (http://cctcc.whu.edu.cn/) and maintained on lyophilized powder. The bacteria were activated, after which the activated strains were inoculated in LB broth and grown overnight (27 °C, 220 R/min). The culture broth was then centrifuged at 6000 rpm for 10 min, and the bacteria were resuspended in phosphate buffer (OD_600_ = 0.8) for later use (Idris et al., 2004).

MWCNTs (98% purity) were purchased from Chengdu Organic Chemicals Co., Ltd., Chinese Academy of Sciences (http://www.cocc.cn/). The inner diameter, outer diameter, and tube length were 2-5 nm, 5-15 nm, and 0.5-2 µm, respectively, and the specific surface area was greater than 350 m^2^/g. The MWCNTs were suspended in ultrapure water to final concentrations of 20 mg/L and 50 mg/L. Afterward, the MWCNT solutions were sonicated at 44 kHz for half an hour in an ultrasonic cleaner for dispersion.

Maize (Hunong No. 101) seeds purchased from the local market were immersed in 75% alcohol for 30 s and 2% sodium hypochlorite for 15 min for sterilization and ultimately washed with ultrapure water three times. The sterilized seeds were subsequently soaked in one of three solutions of MWCNTs at three different concentrations for 20 h before sowing.

The soil used in the experiment was collected from Yanqi Hui Autonomous County, Bayingolin Mongol Autonomous Prefecture, Xinjiang Uygur Autonomous Region (sandy loam with 42.6% sand, 53.3% silt, and 4.1% clay; saturated conductivity, 0.419 dS/m; pH, 7.78; nitrate-nitrogen, 3.36 mg/kg; ammonium nitrogen, 0.16 mg/kg; available potassium, 9.60 mg/kg; and available phosphorus, 10.32 mg/kg).

### 4.2 Experimental design

A pot experiment was conducted in a greenhouse at the agricultural and environmental station of Wuhan University. The conditions included a 13-to-14-h photoperiod of natural daylight and maximum and minimum temperatures of 32 °C and 14 °C, respectively. The experiment was performed in accordance with a completely randomized two-way factorial design representing all combinations of three concentrations of MWCNTs with **B. subtilis* AB 90008* in slightly saline soil. Six treatments were established, each consisting of ten replicates: control (CK), no addition of MWCNTs or *B. subtilis*; M20, addition of 20 mg/L MWCNTs without *B. subtilis*; M50, addition of 50 mg/L MWCNTs without *B. subtilis*; BS, addition of *B. subtilis* without MWCNTs; BM20, addition of 20 mg/L MWCNTs with *B. subtilis*; and BM50, addition of 50 mg/L MWCNTs with *B. subtilis*.

As calculated according to the fertilizer demand for maize growth, 1.92 g of urea, 1.38 g of calcium peroxide, 3 g of potassium sulfate, and 1067 g of air-dried soil were mixed together and added to each pot after the pots were autoclaved twice at 120 °C for 20 min. Four seeds were sown into each pot, and two remained after germination. The seeds were sown on May 7, 2021, and harvested on June 4; thus, the plants grew for 4 weeks in total. During the experiment, all the materials, seeds, soil, and bacterial solutions were prepared and applied as described in Section 4.1.The pots were weighed and irrigated with sterilized water daily to maintain the soil moisture content at approximately 75% of the field capacity. The maize seedlings were irrigated with sodium chloride solutions every two days at the first-leaf stage of maize to induce the salt stress rather than salt shock (Shavrukov, 2013). Each pot was first irrigated with 50 mL of a 150 mmol/L sodium chloride solution, then 50 mL of a 250 mmol/L sodium chloride solution, and finally 50 mL of a 350 mmol/L sodium chloride solution. The salt solution irrigation was stopped when the soil saturated conductivity reached 5.9 dS/m (low-salinity soil resulting in an approximately 50% reduction in maize yield or a 30% decrease in dry matter (Ayers and Westcot, 1976; Katerji et al., 2003)), according to the conversion formula between soil saturated conductivity and total salt content obtained from our experiment: *TDS* = 0.375*EC_e_* – 0.5064 (TDS: soil total salt content and EC_e_: soil saturated conductivity).

Moreover, each seedling from the BS, BM20, and BM50 treatment groups was irrigated with 5 ml of a *B.* s*ubtilis AB 90008* solution (OD_600_ = 0.8), and 5 ml of phosphate buffer were applied to the CK, M20, and M50 treatment groups at the first leaf stage of maize. After one week, each seedling in the BS, BM20, and BM50 treatment groups was irrigated with 5 ml of the *B.* s*ubtilis AB 90008* solution for a second time (5 ml of phosphate buffer each for the remaining groups). At the same time, 5 ml of the MWCNT solution were applied to each plant in the M20, M50, BM20, and BM50 treatment groups (5 ml of sterilized ultrapure water each for the remaining groups).

### 4.3 Agronomic and physiological studies

Agronomic traits such as plant height and dry/fresh weight of maize shoots were measured on June 4, 2021. IAA, ABA, proline, and MDA contents were measured using the double-antibody sandwich ELISA method. The anthrone colorimetric method was performed to determine the soluble sugar content (Eisenhauer et al., 2015). The salicylic acid colorimetric method was performed to determine the nitrate-nitrogen content (Yang et al., 1998). CAT activity was measured using the method reported by Cakmak and Marschner (1992). POD activity was measured using the method reported by Cakmak and Marschner (1992). The Na^+^ and K^+^ contents in the maize leaves and roots were determined by performing flame photometry according to Berry et al. (1946) using a flame photometer (FP6410, INESA, China).

### 4.4 Statistical analysis

R version 4.0.2 was used for data analysis and figure construction. As the nitrate-nitrogen content, POD activity, CAT activity, leaf K^+^ content, and leaf Na^+^/K^+^ ratio did not conform to a normal distribution, Box–Cox transformation was adopted, and the λ value was calculated using maximum likelihood estimation with the power transform function in the car package of R. The transformed and original data that conformed to a normal distribution and whose variance was homogeneous were subjected to one-way and two-way ANOVA. The aov function in R was used for one-way ANOVA, and then the duncan.test function was applied for Duncan’s posttest to judge whether different treatments had significantly different effects on the height, soluble sugar content, IAA content, ABA content, nitrate-nitrogen content, proline content, MDA content, POD activity, CAT activity, Na^+^ and K^+^ contents, and the Na^+^/K^+^ ratio in the leaves and roots of maize. Two-way ANOVA and simple-effect analysis were performed with the MANOVA function in the bruceR package of R. PCA was performed (Leps and Smilauer, 2003) using the prcomp function in R to explore the main differences in the effects of the different treatments.

The growth and development of plants is the comprehensive performance of various physiological and metabolic activities of plants. A correlation analysis was performed to measure the correlation degree between the agronomic traits and other physiological indexes of plants, as well as the correlation degree between other physiological indexes under the experimental conditions, using Spearman’s method. The SEM is a quantitative approach in which systems are considered probabilistic networks to study causal relationships and provide scientific answers and causal understanding (Eisenhauer et al., 2015). An SEM was constructed to quantify and verify the causal relationships between maize plant height, nutrient and ion accumulation, and endogenous hormone contents. Furthermore, based on the SEM, multiple mediating effects were analyzed to partition direct and indirect effects and clarify the multiple pathways by which one entity may influence another (Zhang et al., 2017b). The SEM was constructed using the sem function of the lavaan package in R. Given the small amount of data and the fact that some of the data did not conform to a normal distribution, the nonparametric percentile bootstrapping method was applied in our SEM (Preacher and Hayes, 2004). The maximum likelihood (χ^2^), CFI, and RMSEA were used to examine the fitness of the SEM.

## 5 Conclusions

A pot experiment was conducted to investigate the potential of MWCNTs and *B. subtilis* to combat salt stress in maize plants. The dose-dependent effects of MWCNTs were confirmed: 20 mg/L MWCNTs promoted the accumulation of osmolytes in maize, particularly K^+^ in the leaves and roots through a process mediated by ABA, but the above-mentioned promoting effect decreased significantly in 50 mg/L MWCNTs-treated plants. The increased lipid peroxidation and antioxidant activities suggested that MWCNTs induce oxidative stress in maize growing in low-salinity soils and that *B. subtilis* reduced the oxidative stress caused by MWCNTs, as indicated by the lower MDA content. Notably, the addition of MWCNTs at all concentrations hastened leaf Na^+^ accumulation, exacerbating the Na^+^/K^+^ imbalance, but this effect was completely reversed after the addition of **B. subtilis*.* The combination of 50 mg/L MWCNTs and *B. subtilis* induced an osmotic adjustment in maize by increasing osmolytes and regulated the ion balance by decreasing the leaf Na^+^/K^+^ ratio through the mediating effects of ABA and nitrate, maximizing the height and dry/fresh weight of maize. Taken together, the results of this study indicated that *B. subtilis* and MWCNTs synergistically improved maize salt tolerance.

## Data availability statement

The original contributions presented in the study are included in the article/**Supplementary Material**. Further inquiries can be directed to the corresponding authors.

## Author contributions

Conceptualization, WZ and GL; Data curation, YL; Funding acquisition, WZ; Investigation, YL; Methodology, WZ, YH and AS; Supervision, WZ; Visualization, YL and YH; Writing – original draft, YL; Writing – review & editing, WZ, GL, CA, HC, TG and AS. All authors contributed to the article and approved the submitted version.

## References

[B1] Acosta-Motos J. R. Ortuno M. F. Bernal-Vicente A. Diaz-Vivancos P. Sanchez-Blanco M. J. Hernandez J. A. (2017). Plant responses to salt stress: Adaptive mechanisms. Agronomy-Basel 7 (1), 18. doi: 10.3390/agronomy7010018

[B2] Ahmed B. Syed A. Rizvi A. Shahid M. Bahkali A. H. Khan M. S.. (2021). Impact of metal-oxide nanoparticles on growth, physiology and yield of tomato (Solanum lycopersicum l.) modulated by azotobacter salinestris strain ASM. Environ. pollut. 269, 116218. doi: 10.1016/j.envpol.2020.116218 33316490

[B3] Akhtyamova Z. Arkhipova T. Martynenko E. Nuzhnaya T. Kuzmina L. Kudoyarova G.. (2021). Growth-promoting effect of rhizobacterium (Bacillus subtilis IB22) in salt-stressed barley depends on abscisic acid accumulation in the roots. Int. J. Mol. Sci. 22 (19), 10680. doi: 10.3390/ijms221910680 34639021 PMC8508976

[B4] Anwar Hossain M. Hoque M. A. Burritt D. J. Fujita M. (2014). “Chapter 16 - proline protects plants against abiotic oxidative stress: Biochemical and molecular mechanisms,” in Oxidative damage to plants. Ed. Ahmad P. (San Diego: Academic Press), 477–522.

[B5] Ashwini N. Srividya S. (2014). Potentiality of bacillus subtilis as biocontrol agent for management of anthracnose disease of chilli caused by colletotrichum gloeosporioides OGC1. Biotech. 4 (2), 127–136. doi: 10.1007/s13205-013-0134-4 PMC3964249 28324440

[B6] Ayers R. S. Westcot D. W. (1976). Water quality for agriculture (Rome: Food and Agriculture Organization of the United Nations).

[B7] Barnawal D. Bharti N. Pandey S. S. Pandey A. Chanotiya C. S. Kalra A. (2017). Plant growth-promoting rhizobacteria enhance wheat salt and drought stress tolerance by altering endogenous phytohormone levels and TaCTR1/TaDREB2 expression. Physiol. Plantarum 161 (4), 502–514. doi: 10.1111/ppl.12614 28786221

[B8] Berry J. W. Chappell D. G. Barnes R. B. (1946). Improved method of flame photometry. Ind. Eng. Chem. Analytical Ed. 18 (1), 19–24. doi: 10.1021/i560149a005

[B9] Cakmak I. Marschner H. (1992). Magnesium-deficiency and high light-intensity enhance activities of superoxide-dismutase, ascorbate peroxidase, and glutathione-reductase in bean-leaves. Plant Physiol. 98 (4), 1222–1227. doi: 10.1104/pp.98.4.1222 16668779 PMC1080336

[B10] Chen S. Li J. Wang S. Hüttermann A. Altman A. (2001). Salt, nutrient uptake and transport, and ABA of populus euphratica; a hybrid in response to increasing soil NaCl. Trees 15 (3), 186–194. doi: 10.1007/s004680100091

[B11] Chen L. Yang J. Li X. Liang T. Nie C. Xie F.. (2020). Carbon nanoparticles enhance potassium uptake *via* upregulating potassium channel expression and imitating biological ion channels in BY-2 cells. J. Nanobiotechnol. 18 (1), 21. doi: 10.1186/s12951-020-0581-0 PMC6986061 31992314

[B12] Chhipa H. Joshi P. (2016). “Nanofertilisers, nanopesticides and nanosensors in agriculture,” in Nanoscience in food and agriculture 1 (Cham, Switzerland: Springer), 247–282.

[B13] Chu L.-l. Kang Y.-H. Wan S.-Q. (2016). Effect of different water application intensity and irrigation amount treatments of microirrigation on soil-leaching coastal saline soils of north China. J. Integr. Agric. 15 (9), 2123–2131. doi: 10.1016/s2095-3119(15)61263-1

[B14] Coskun D. Britto D. T. Kronzucker H. J. (2017). The nitrogen–potassium intersection: membranes, metabolism, and mechanism. J. P. Cell Environ. 40 (10), 2029–2041. doi: 10.1111/pce.12671 26524711

[B15] Dai J. L. Duan L. S. Dong H. Z. (2015). Comparative effect of nitrogen forms on nitrogen uptake and cotton growth under salinity stress. J. Plant Nutr. 38 (10), 1530–1543. doi: 10.1080/01904167.2014.983126

[B16] Eisenhauer N. Bowker M. A. Grace J. B. Powell J. R. (2015). From patterns to causal understanding: Structural equation modeling (SEM) in soil ecology. Pedobiologia 58 (2-3), 65–72. doi: 10.1016/j.pedobi.2015.03.002

[B17] Fang X. Z. Liu X. X. Zhu Y. X. Ye J. Y. Jin C. W. J. B. (2020). K+ uptake and root-to-shoot allocation in arabidopsis require coordination of nitrate transporter1/peptide transporter family member NPF6. 3/NRT1 1, 674903. doi: 10.1101/674903

[B18] Ghanbari F. Nasarzadeh P. Seydi E. Ghasemi A. Joghataei M. T. Ashtari K.. (2017). Mitochondrial oxidative stress and dysfunction induced by single- and multiwall carbon nanotubes: A comparative study. J. Biomed. Mater. Res. Part A. 105 (7), 2047–2055. doi: 10.1002/jbm.a.36063 28296041

[B19] Gohari G. Safai F. Panahirad S. Akbari A. Rasouli F. Dadpour M. R.. (2020). Modified multiwall carbon nanotubes display either phytotoxic or growth promoting and stress protecting activity in ocimum basilicum l. in a concentration-dependent manner. Chemosphere 249, 126171. doi: 10.1016/j.chemosphere.2020.126171 32087452

[B20] Hafeez M. B. Zahra N. Zahra K. Raza A. Khan A. Shaukat K.. (2021). Brassinosteroids: Molecular and physiological responses in plant growth and abiotic stresses. Plant Stress 2, 100029. doi: 10.1016/j.stress.2021.100029

[B21] Hartono M. R. Kushmaro A. Chen X. Marks R. S. (2018). Probing the toxicity mechanism of multiwalled carbon nanotubes on bacteria. Environ. Sci. pollut. Res. Int. 25 (5), 5003–5012. doi: 10.1007/s11356-017-0782-8 29209964

[B22] Hatami M. Hadian J. Ghorbanpour M. (2017). Mechanisms underlying toxicity and stimulatory role of single-walled carbon nanotubes in hyoscyamus niger during drought stress simulated by polyethylene glycol. J. Hazard Mater. 324 (Pt B), 306–320. doi: 10.1016/j.jhazmat.2016.10.064 27810325

[B23] He T. Cramer G. R. (1996). Abscisic acid concentrations are correlated with leaf area reductions in two salt-stressed rapid-cycling brassica species. Plant Soil 179 (1), 25–33. doi: 10.1007/Bf00011639

[B24] Hidri R. Metoui-Ben Mahmoud O. Farhat N. Cordero I. Pueyo J. J. Debez A.. (2019). Arbuscular mycorrhizal fungus and rhizobacteria affect the physiology and performance of Sulla coronaria plants subjected to salt stress by mitigation of ionic imbalance. J. Plant Nutr. Soil Sci. 182 (3), 451–462. doi: 10.1002/jpln.201800262

[B25] Hong M. Gong J. L. Cao W. C. Fang R. Cai Z. Ye J.. (2022). The combined toxicity and mechanism of multi-walled carbon nanotubes and nano zinc oxide toward the cabbage. Environ. Sci. pollut. Res. Int. 29 (3), 3540–3554. doi: 10.1007/s11356-021-15857-4 34389955

[B26] Hu Y. Zhang P. Zhang X. Liu Y. Feng S. Guo D.. (2021). Multi-wall carbon nanotubes promote the growth of maize (Zea mays) by regulating carbon and nitrogen metabolism in leaves. J. Agric. Food Chem. 69 (17), 4981–4991. doi: 10.1021/acs.jafc.1c00733 33900073

[B27] Idris E. E. Bochow H. Ross H. Borriss R. (2004). Use of bacillus subtilis as biocontrol agent. VI. phytohormone-like action of culture filtrates prepared from plant growth-promoting bacillus amyloliquefaciens FZB24, FZB42, FZB45 and bacillus subtilis FZB37. Z. Fur. Pflanzenkrankheiten Und Pflanzenschutz-J. Plant Dis. Prot. 111 (6), 583–597.

[B28] Jaenicke H. Lips H. S. Ullrich W. R. (1996). Growth, ion distribution, potassium and nitrate uptake of leucaena leucocephala, and effects of NaCl. Plant Physiol. Biochem. 34 (5), 743–751.

[B29] Jiang H. S. Li M. Chang F. Y. Li W. Yin L. Y. (2012). Physiological analysis of silver nanoparticles and AgNO3 toxicity to spirodela polyrhiza. Environ. Toxicol. Chem. 31 (8), 1880–1886. doi: 10.1002/etc.1899 22639346

[B30] Joshi A. Sharma L. Kaur S. Dharamvir K. Nayyar H. Verma G. (2020). Plant nanobionic effect of multi-walled carbon nanotubes on growth, anatomy, yield and grain composition of rice. BioNanoScience 10 (2), 430–445. doi: 10.1007/s12668-020-00725-1

[B31] Kader M. A. Seidel T. Golldack D. Lindberg S. (2006). Expressions of OsHKT1, OsHKT2, and OsVHA are differentially regulated under NaCl stress in salt-sensitive and salt-tolerant rice (Oryza sativa l.) cultivars. J. Exp. Bot. 57 (15), 4257–4268. doi: 10.1093/jxb/erl199 17088362

[B32] Karami A. Sepehri A. (2018). Beneficial role of MWCNTs and SNP on growth, physiological and photosynthesis performance of barley under NaCl stress. J. Soil Sci. Plant Nutr. 18, 752–771. doi: 10.4067/S0718-95162018005002202

[B33] Katerji N. van Hoorn J. W. Hamdy A. Mastrorilli M. (2003). Salinity effect on crop development and yield, analysis of salt tolerance according to several classification methods. Agric. Water Manage. 62 (1), 37–66. doi: 10.1016/s0378-3774(03)00005-2

[B34] Koul O. (2019). Nano-biopesticides today and future perspectives (London: Academic Press, an imprint of Elsevier).

[B35] Kroeger J. H. Zerzour R. Geitmann A. (2011). Regulator or driving force? the role of turgor pressure in oscillatory plant cell growth. PloS One 6 (4), 18549. doi: 10.1371/journal.pone.0018549 PMC3081820 21541026

[B36] Leps J. Smilauer P. (2003). Multivariate analysis of ecological data using CANOCO (Cambridge, UK; New York: Cambridge University Press).

[B37] Lin D. Xing B. (2007). Phytotoxicity of nanoparticles: inhibition of seed germination and root growth. Environ. pollut. 150 (2), 243–250. doi: 10.1016/j.envpol.2007.01.016 17374428

[B38] Li H. Yue H. Li L. Liu Y. Zhang H. Wang J.. (2021). Seed biostimulant bacillus sp. MGW9 improves the salt tolerance of maize during seed germination. AMB Express 11 (1), 74. doi: 10.1186/s13568-021-01237-1 34032933 PMC8149540

[B39] Martinez-Ballesta M. C. Zapata L. Chalbi N. Carvajal M. (2016). Multiwalled carbon nanotubes enter broccoli cells enhancing growth and water uptake of plants exposed to salinity. J. Nanobiotechnol. 14 (1), 42. doi: 10.1186/s12951-016-0199-4 PMC4898372 27278384

[B40] Mastronardi E. Tsae P. Zhang X. Monreal C. DeRosa M. C. (2015). “Strategic role of nanotechnology in fertilizers: Potential and limitations,” in Nanotechnologies in food and agriculture (Cham, Switzerland: Springer), 25–67.

[B41] Medeiros C. A. A. Bettiol W. (2021). Multifaceted intervention of bacillus spp. against salinity stress and fusarium wilt in tomato. J. Appl. Microbiol. 131 (5), 2387–2401. doi: 10.1111/jam.15095 33817910

[B42] Mondal A. Basu R. Das S. Nandy P. (2011). Beneficial role of carbon nanotubes on mustard plant growth: an agricultural prospect. J. Nanoparticle Res. 13 (10), 4519–4528. doi: 10.1007/s11051-011-0406-z

[B43] Monreal C. M. DeRosa M. Mallubhotla S. C. Bindraban P. S. Dimkpa C. (2015). Nanotechnologies for increasing the crop use efficiency of fertilizer-micronutrients. Biol. Fertil. Soils 52 (3), 423–437. doi: 10.1007/s00374-015-1073-5

[B44] Munns R. Gilliham M. (2015). Salinity tolerance of crops - what is the cost? New Phytol. 208 (3), 668–673. doi: 10.1111/nph.13519 26108441

[B45] Mustafa G. Akhtar M. S. Abdullah R. (2019). “Global concern for salinity on various agro-ecosystems,” in Salt stress, microbes, and plant interactions: Causes and solution (Singapore: Springer), 1–19.

[B46] Nakano M. M. Zuber P. (1998). Anaerobic growth of a “Strict aerobe”. (BACILLUS SUBTILIS) 52 (1), 165–190. doi: 10.1146/annurev.micro.52.1.165 9891797

[B47] Preacher K. J. Hayes A. F. (2004). SPSS And SAS procedures for estimating indirect effects in simple mediation models. Behav. Res. Methods Instruments Comput. 36 (4), 717–731. doi: 10.3758/Bf03206553 15641418

[B48] Rahmani N. Radjabian T. Soltani B. M. (2020). Impacts of foliar exposure to multi-walled carbon nanotubes on physiological and molecular traits of salvia verticillata l., as a medicinal plant. Plant Physiol. Biochem. 150, 27–38. doi: 10.1016/j.plaphy.2020.02.022 32109787

[B49] Ramesh A. Sharma S. K. Sharma M. P. Yadav N. Joshi O. P. (2014). Inoculation of zinc solubilizing bacillus aryabhattai strains for improved growth, mobilization and biofortification of zinc in soybean and wheat cultivated in vertisols of central India. Appl. Soil Ecol. 73, 87–96. doi: 10.1016/j.apsoil.2013.08.009

[B50] Rao E. L. N. Sharma P. C. (1995). Alleviation of salinity stress in chickpea by rhizobium inoculation or nitrate supply. Biol. Plantarum 37 (3), 405–410. doi: 10.1007/BF02913989

[B51] Rong-fa L. Peng L. Shu-ting D. Ji-wang Z. Bin Z. (2019). Effect of combined application of fertilizer and bacillus subtilis on grain yield and fertilizer utilization efficiency of summer maize (Zea mays l.). J. Plant Nutr. Fertilizers 25, 1607–1614. doi: 10.11674/zwyf.18344

[B52] Saikia J. Sarma R. K. Dhandia R. Yadav A. Bharali R. Gupta V. K.. (2018). Alleviation of drought stress in pulse crops with ACC deaminase producing rhizobacteria isolated from acidic soil of northeast India (vol 8, 2018). Sci. Rep. 8, 560. doi: 10.1038/s41598-018-25174-5 29476114 PMC5824784

[B53] SAINAO W. CAO J. PANG H. SHI Z. FENG H. (2020). Multi-walled carbon nanotubes: their effects on the physiological responses of oryza sativa l. seedlings and the toxicity of trichlorobenzene. Chin. J. Appl. Environ. Biol. 26, 534–542. doi: 10.19675/j.cnki.1006-687x.2019.07037

[B54] Saito S. Uozumi N. (2019). Guard cell membrane anion transport systems and their regulatory components: An elaborate mechanism controlling stress-induced stomatal closure. Plants-Basel 8 (1), 9. doi: 10.3390/plants8010009 30609843 PMC6359458

[B55] Saleem S. Iqbal A. Ahmed F. Ahmad M. (2021). Phytobeneficial and salt stress mitigating efficacy of IAA producing salt tolerant strains in gossypium hirsutum. Saudi J. Biol. Sci. 28 (9), 5317–5324. doi: 10.1016/j.sjbs.2021.05.056 34466110 PMC8381066

[B56] Sarma B. K. Yadav S. K. Singh D. P. Singh H. B. (2012). “Rhizobacteria mediated induced systemic tolerance in plants: Prospects for abiotic stress management,” in Bacteria in agrobiology: Stress management (Berlin, Germany: Springer), 225–238.

[B57] Seddighinia F. S. Iranbakhsh A. Oraghi Ardebili Z. Nejad Satari T. Soleimanpour S. (2019). Seed priming with cold plasma and multi-walled carbon nanotubes modified growth, tissue differentiation, anatomy, and yield in bitter melon (Momordica charantia). J. Plant Growth Regul. 39 (1), 87–98. doi: 10.1007/s00344-019-09965-2

[B58] Shang Y. Hasan M. K. Ahammed G. J. Li M. Yin H. Zhou J. (2019). Applications of nanotechnology in plant growth and crop protection: A review. Molecules 24 (14), 2558. doi: 10.3390/molecules24142558 31337070 PMC6680665

[B59] Shavrukov Y. (2013). Salt stress or salt shock: which genes are we studying? J. Exp. Bot. 64 (1), 119–127. doi: 10.1093/jxb/ers316 23186621

[B60] Shkolnik-Inbar D. Adler G. Bar-Zvi D. (2013). ABI4 downregulates expression of the sodium transporter HKT1;1 in arabidopsis roots and affects salt tolerance. Plant J. 73 (6), 993–1005. doi: 10.1111/tpj.12091 23240817

[B61] Shweta V. K. Sharma S. Narayan R. P. Srivastava P. Khan A. S. Nawal K. D.. (2017). “Plants and carbon nanotubes (CNTs) interface: Present status and future prospects,” in Nanotechnology (Singapore: Springer), 317–340.

[B62] Sun B. Gu L. K. Bao L. J. Zhang S. W. Wei Y. X. Bai Z. H.. (2020). Application of biofertilizer containing bacillus subtilis reduced the nitrogen loss in agricultural soil. Soil Biol. Biochem. 148, 107911. doi: 10.1016/j.soilbio.2020.107911

[B63] Tiwari D. K. Dasgupta-Schubert N. Villaseñor Cendejas L. M. Villegas J. Carreto Montoya L. Borjas García S. E. (2013). Interfacing carbon nanotubes (CNT) with plants: enhancement of growth, water and ionic nutrient uptake in maize (Zea mays) and implications for nanoagriculture. Appl. Nanosci. 4 (5), 577–591. doi: 10.1007/s13204-013-0236-7

[B64] Upadhyay S. K. Singh J. S. Singh D. P. (2011). Exopolysaccharide-producing plant growth-promoting rhizobacteria under salinity condition. Pedosphere 21 (2), 214–222. doi: 10.1016/S1002-0160(11)60120-3

[B65] Vithanage M. Seneviratne M. Ahmad M. Sarkar B. Ok Y. S. (2017). Contrasting effects of engineered carbon nanotubes on plants: a review. Environ. Geochem Health 39 (6), 1421–1439. doi: 10.1007/s10653-017-9957-y 28444473

[B66] Wang R. Tischner R. Gutiérrez R. A. Hoffman M. Xing X. Chen M.. (2004). Genomic analysis of the nitrate response using a nitrate reductase-null mutant of arabidopsis Plant Physiol. 136, 1, 2512–2522. doi: 10.1104/pp.104.044610 15333754 PMC523318

[B67] Woo O. G. Kim H. Kim J. S. Keum H. L. Lee K. C. Sul W. J.. (2020). Bacillus subtilis strain GOT9 confers enhanced tolerance to drought and salt stresses in arabidopsis thaliana and brassica campestris. Plant Physiol. Biochem. 148, 359–367. doi: 10.1016/j.plaphy.2020.01.032 32018064

[B68] Wu M.-y. Ruo-chao H. Xiao-hai T. Xiao-ling W. Guo-hui M. Hai-tao T. J. H. R. (2010). Effect of adding nano-carbon in slow release fertilizer on grain yield and nitrogen use efficiency of super hybrid rice Hybrid Rice 4, 034. doi: 10.4028/www.scientific.net/AMM.37-38.1549

[B69] Yang J. E. Skogley E. O. Schaff B. E. Kim J. J. (1998). A simple spectrophotometric determination of nitrate in water, resin, and soil extracts. Soil Sci. Soc. America J. 62 (4), 1108–1115. doi: 10.2136/sssaj1998.03615995006200040036x

[B70] Yang L. Zhou B. Hou Y. Wang Q. And Chen X. (2021). Effects of bacillus subtilis on growth of winter wheat and distribution of soil water and nitrogen under salt stress. J. Drainage Irrigation Machinery Eng. 39, 517–524. doi: 10.3969/j.issn.1674.8530.19.0267

[B71] Zhang Y. Fang J. Wu X. Dong L. (2018). Na(+)/K(+) balance and transport regulatory mechanisms in weedy and cultivated rice (Oryza sativa l.) under salt stress. BMC Plant Biol. 18 (1), 375. doi: 10.1186/s12870-018-1586-9 30594151 PMC6311050

[B72] Zhang H. Kim M.-S. Sun Y. Dowd S. E. Shi H. Paré P. W. (2008). Soil bacteria confer plant salt tolerance by tissue-specific regulation of the sodium transporter HKT1. Mol. Plant-Microbe Interact. 21, 6, 737–744. doi: 10.1094/MPMI-21-6-0737 18624638

[B73] Zhang D. Y. Li J. N. Niu X. Deng C. Y. Song X. H. Li W. X.. (2021). GhANN1 modulates the salinity tolerance by regulating ABA biosynthesis, ion homeostasis and phenylpropanoid pathway in cotton. Environ. Exp. Bot. 185, 104427. doi: 10.1016/j.envexpbot.2021.104427

[B74] Zhang X. Wang T. Liu M. Sun W. Zhang W.-H. (2019). Calmodulin-like gene MtCML40 is involved in salt tolerance by regulating MtHKTs transporters in medicago truncatula. Environ. Exp. Bot. 157, 79–90. doi: 10.1016/j.envexpbot.2018.09.022

[B75] Zhang W. Wang C. Lu T. Zheng Y. (2017b). Cooperation between arbuscular mycorrhizal fungi and earthworms promotes the physiological adaptation of maize under a high salt stress. Plant Soil 423 (1-2), 125–140. doi: 10.1007/s11104-017-3481-9

[B76] Zhang H. Yue M. Zheng X. Xie C. Zhou H. Li L. (2017a). Physiological effects of single- and multi-walled carbon nanotubes on rice seedlings. IEEE Trans. Nanobiosci. 16 (7), 563–570. doi: 10.1109/TNB.2017.2715359 28622672

[B77] Zhao G. Zhao Y. Lou W. Su J. Wei S. Yang X.. (2019). Nitrate reductase-dependent nitric oxide is crucial for multi-walled carbon nanotube-induced plant tolerance against salinity. Nanoscale 11 (21), 10511–10523. doi: 10.1039/c8nr10514f 31116204

