# Supplementary material for: The effects of multiwalled carbon nanotubes and Bacillus subtilis treatments on the salt tolerance of maize seedlings
Source: Front Plant Sci. 2022 Dec 9;13:1093529. doi: 10.3389/fpls.2022.1093529 (PMC9780592; doi:10.3389/fpls.2022.1093529)
Supplement: Supplementary file 1 [file Table_1.docx]

Supplementary Material

# Supplementary Tables

**Supplementary Table 1**. Two-way ANOVA (* = p < 0.05, ** = p < 0.01, and *** = p < 0.001).

| Factors | MDA  (nmol/g FW) | Proline  (ng/g FW) | POD  (U/g FW) | CAT  (U/g FW) | Leaf K+  (mg/g DW) | Root K+  (mg/g DW) | Leaf Na+  (mg/g DW) | Root Na+  (mg/g DW) | Leaf Na+/K+ | Root Na+/K+ |
| --- | --- | --- | --- | --- | --- | --- | --- | --- | --- | --- |
| B. subtilis | 9.59** | 16.74*** | 78.81*** | 30.81*** | 1.56 | 141.63*** | 1523.10*** | 0.00 | 3355.36*** | 6.17 |
| MWCNTs | 23.80*** | 36.72*** | 0.93 | 24.58*** | 11.20** | 56.38*** | 109.76*** | 0.08 | 956.77*** | 2.96 |
| B. subtilis × MWCNTs | 1.26 | 10.37*** | 34.39*** | 5.18* | 15.75*** | 288.57*** | 276.22*** | 8.99** | 1029.15*** | 14.63*** |
